# Neurochemical and Genetic Alterations in Chronic Stimulant (Crystal and Captagon) Users: A Comparative Analysis of Monoamine Biomarkers and Addiction‐Related Gene Expression

**DOI:** 10.1155/bri/2398129

**Published:** 2026-05-08

**Authors:** Yasameen Hameed Jasim, Basma A. Al-Mashhdani, Mustafa Abd-Almajeed Abd-Alkareem, Ashfaq Ahmad Shah Bukhari, Rasha Hameed Jasim, Sonia Tabasum, Lala Hasanli, Rajwali Khan

**Affiliations:** ^1^ Department of Applied Chemistry, College of Applied Sciences, Samarra University, Samarra, 34010, Iraq; ^2^ Department of Chemistry, College of Education, Al-Iraqia University, Baghdad, Iraq, aliraqia.edu.iq; ^3^ Department of Pathological Analysis, College of Applied Sciences, Samarra University, Samarra, 34010, Iraq; ^4^ Department of Physiology, RAK College of Medical Sciences, Ras Al Khaimah Medical and Health Sciences University, Ras Al-Khaimah, UAE; ^5^ Department of Biochemistry, University of Agriculture Faisalabad, Faisalabad, 38000, Pakistan, uaf.edu.pk; ^6^ Department of Basic Medical Sciences, Faculty of Medicine, Nakhchivan State University, Nakhchivan, AZ7012, Azerbaijan, ndu.edu.az; ^7^ National Water and Energy Center, United Arab Emirates University, Al Ain, 15551, UAE, uaeu.ac.ae

**Keywords:** CARTPT, dopamine, gene expression, neurotransmitters, SLC6A3, stimulant use disorder

## Abstract

This investigation examined the long‐term impact of stimulant use, assessing systematic dysfunction of the nervous system and how these changes were reflected in the gene expression of two addiction‐related genes, SLC6A3 (dopamine transporter) and CARTPT (cocaine‐ and amphetamine‐regulated transcript). Study subjects included 100 men over the age of 18 who have used stimulants (crystal and Captagon) as well as a group of 30 men of the same age who were healthy. Blood samples were taken from each subject, and plasma levels of dopamine, serotonin, norepinephrine, and total protein were measured using enzyme‐linked immunosorbent assay (ELISA). Gene expression was analyzed in peripheral blood mononuclear cells (PBMCs) using quantitative reverse transcription–polymerase chain reaction (qRT‐PCR). Relative expression of SLC6A3 and CARTPT was calculated using the 2^−ΔΔCt^ method with GAPDH serving as an internal control. Stimulant users had significantly higher concentrations of plasma dopamine and norepinephrine compared to non‐users, while also having much lower levels of both plasma serotonin and total protein compared to controls (*p* < 0.0001). qRT‐PCR showed that the expression of SLC6A3 and CARTPT was significantly increased in the stimulant group (5–7‐fold increase). These findings, which reflect both biochemical and gene expression level changes, indicate increased dopaminergic activity, decreased serotonergic activity, and changes in the neuropeptide pathways related to stress and reward due to chronic use of stimulants. Overall, the combination of the biochemical and gene expression data indicates that chronic crystal and Captagon users demonstrate significant systemic dysfunction. The significant changes in the levels of monoamines and the activation of the SLC6A3 and CARTPT genes indicate that these two genes could serve as peripheral biomarkers in assessing stimulant use disorder. These data give a mechanistic basis for developing new diagnostic and treatment methods based on dopaminergic and neuropeptide signaling pathways involved in the use of stimulants.

## 1. Introduction

Stimulant use disorder (SUD)is a neurochemical condition that results from prolonged use of stimulant drugs (i.e., those that activate the brain’s “pleasure center”) such as methamphetamine and/or other amphetamine‐type substances (i.e., crystal meth and Captagon) [1]. These types of substances produce an increase in the concentration of three major neurotransmitters in the brain (dopamine, norepinephrine, and serotonin) [2, 3] by causing those neurotransmitters to be released into the synapse and preventing them from being reabsorbed back into the nerve cells (this is the action that produces the pleasurable sensations). This can lead to elevated levels of emotional and physical activity in the short term, but over time, long‐term use can lead to changes in the structure and function of the brain, leading to dependence, overt and covert changes in psychological functioning, and can produce neurotoxic effects [1, 4].

The criteria for diagnosing SUDs as defined by the Diagnostic and Statistical Manual (DSM), fifth edition, and the International Classification of Diseases, 11th edition, are impaired control over use of the substance, persistent cravings for the substance, withdrawal symptoms from the substance, and continued use of the substance despite evidence that it is harmful. The use of stimulants as substances of abuse has been increasing globally, according to the United Nations Office on Drugs and Crime (UNODC) report, with methamphetamine abuse increasing in both developed countries and low‐income countries. Some of the health consequences of abusing stimulants include cardiovascular disease, cognitive impairment, psychosis, aggression toward others, and early death [4, 5].

Exposure to stimulants over a long period of time changes the way neurotransmitters work in the brain, especially dopamine, which leads to fewer available receptors, less effective transporter functioning, and altered rates of neurotransmitter release and reuptake [6]. These changes create a long‐term cycle of addiction that is characterized by tolerance, compulsive drug‐seeking behaviors, and withdrawal‐induced negative emotional states. Other neurotransmitter systems have also been impacted by chronic stimulant use, including serotonin and norepinephrine pathways, which are thought to contribute to feelings of anxiety, emotional dysregulation, and increased sensitivity to stress among those who have been affected. Finally, genetic factors may also influence the development and persistence of stimulant addiction; two of the most notable genes with regard to addiction are SLC6A3 and cocaine‐ and amphetamine‐ regulated transcript (CARTPT). The SLC6A3 gene provides instructions for the manufacture of the dopamine transporter (DAT) protein, which serves to recycle dopamine that was released into the synapse during dopamine‐related events or when dopamine is absent after transportation out of the synapse. Changes in the expression level of the gene may increase both duration and intensity of dopaminergic activity, which may promote addiction, by promoting behaviors that increase the likelihood the individual will pursue further substance exposure [7–9]. The CARTPT gene is responsible for producing a neuropeptide known as the cocaine‐ and amphetamine‐regulated transcript (CART), which is responsible for regulating reward systems, responding to stress, and controlling appetite. The level of expression for the CARTPT gene is associated with the degree of craving and relapse susceptibility in individuals who are dependent on stimulants [10]. A recent advance in molecular biology technologies, particularly quantitative reverse transcription–polymerase chain reaction (qRT‐PCR), has allowed researchers to accurately quantify the effect of various drugs on gene expression. qRT‐PCR maintains a high level of specificity and allows for the quantification of mRNA transcripts; therefore, researchers can have a thorough understanding of the molecular changes caused by repeated stimulant exposure. By using qRT‐PCR in conjunction with biochemical measures of peripheral biomarkers, such as dopamine or serotonin levels or levels of norepinephrine or plasma proteins, researchers will obtain an overall measure of neurochemical and genetic changes resulting from SUDs [11].

The goal of the present study was to examine the two‐fold nature of biochemical and genetic changes in individuals who use stimulants on a chronic basis in comparison to healthy controls. To this end, we measured plasma levels of dopamine, serotonin, norepinephrine, and total protein in combination with expression levels of SLC6A3 [12, 13] and CARTPT, both of which have been shown to have close relationships to dopaminergic signaling and the control of reward. The methodology of the current study was designed around several core research questions. First, did the plasma levels of dopamine, serotonin, and norepinephrine differ significantly between individuals who chronically used stimulants compared to the matched controls? Second, is there a difference in total plasma protein levels between the groups? If so, these differences may be indicative of more generalized systemic impacts of stimulant exposure. Third, did the gene expression data from SLC6A3 and CARTPT correspond to the observed differences in neurotransmitter levels? In an investigation involving 100 men with a long history of using stimulants and also a control group of 30 men of comparable age without a history of stimulant use, both blood samples were obtained to determine the plasma levels of dopamine, serotonin, norepinephrine, and total side proteins using the enzyme‐linked immunesorbent assay (ELISA) as well as isolating and extracting RNA from isolated peripheral blood mononuclear cells (PBMCs) to perform RNA expression (gene expression) by qRT‐PCR and measure RNA expression compared to the house‐keeping gene (GAPDH) with the use of the 2^−ΔΔCt^ method for comparing gene expression across the different subject groups. This investigation has been performed in order to explore the biochemical and genetic changes that occur with stimulant use as well as to further expand our overall understanding of the biological mechanisms involved in SUD that may lead to identifying biomarkers that may one day aid the development of new diagnostic and treatment methods. Throughout this report, the term SUD has been used as per the DSM‐5 diagnostic criteria for SUDs.

## 2. Material and Method

### 2.1. Sample Collection

A total of 130 participants were enrolled in this case–control study, including 100 individuals with SUD and 30 healthy controls. Participants were aged between 18 and 35 years (mean ± standard deviation [SD]: 26.4 ± 4.8 years). The study population consisted predominantly of male participants, reflecting the demographic distribution of stimulant use in the study setting.

SUD was defined according to the Diagnostic and Statistical Manual of Mental Disorders, Fifth Edition (DSM‐5) criteria, based on clinical history and self‐reported patterns of stimulant use. All individuals in the stimulant group reported regular use of crystal methamphetamine and/or Captagon for a minimum duration of 12 months prior to enrollment, with reported use ranging from one to several years.

Participants were screened for polysubstance use using structured interviews. Individuals with alcohol use disorder, regular alcohol consumption (> 2 standard drinks/day), cannabis use disorder, opioid use, or use of other illicit substances were excluded. Occasional social alcohol consumption (< 2 drinks/week) was permitted but documented. Smoking status was recorded but was not used as an exclusion criterion.

Inclusion criteria for the stimulant group included confirmed chronic stimulant use and absence of acute medical illness at the time of participation. Exclusion criteria included a history of major neurological disorders, chronic systemic diseases, or current use of medications known to influence monoaminergic neurotransmission, including antidepressants, antipsychotics, and beta‐adrenergic blockers.

Control participants were recruited from the general population and had no lifetime history of SUD, alcohol use disorder, or other SUDs, as assessed through structured interviews. Controls were free from psychiatric illness and were not receiving medications known to affect monoaminergic neurotransmission. Psychiatric comorbidities were evaluated through structured clinical interviews conducted by trained clinicians. Participants with major depressive disorder, bipolar disorder, schizophrenia, anxiety disorders requiring pharmacological treatment, or other severe psychiatric illnesses were excluded.

Venous blood samples were collected in the morning (08:00–10:00) under fasting conditions. For participants in the stimulant group, blood sampling was performed at least 48 h after the last reported stimulant use to minimize the influence of acute intoxication. All participants were clinically stable at the time of sampling.

The study protocol was approved by the Institutional Ethics Committee of Samarra University (Approval No. 1126), and written informed consent was obtained from all participants prior to enrollment.

### 2.2. Biochemical Analysis (ELISA)

The plasma levels of the monoamines dopamine, serotonin, and norepinephrine were all determined using ELISA purchased from commercial vendors. All venous blood samples for determination of the above monoamines and total protein were collected in tubes containing EDTA, were centrifuged for 10 min at 3000 RPM at 4°C, and plasma aliquots were stored at −80°C until assay. The levels of dopamine and norepinephrine in plasma were measured using the Dopamine and Noradrenaline High Sensitivity ELISA kit (Eagle Bio, USA), and the level of serotonin in plasma was measured using the Human Serotonin ELISA kit (FineTest, EH4005, China). The plasma levels of total protein were determined by routine means using standard colorimetric assays. Duplicates of all samples in both the SUD group (*n* = 100) and the control group (*n* = 30) were assayed. The optical density at 450 nm was measured using a microplate reader, and the concentrations of each analyte were calculated based on standard curves developed specifically for each plate. Intra‐assay and inter‐assay coefficients of variation were each maintained at less than 10%. Individual representative biochemical values for participants (*n* = 130) can be found in Supplemental Table S4. Group‐level summary statistics for plasma monoamine and total protein concentrations for chronic crystal and Captagon users compared with healthy controls are shown in Table 1.

**TABLE 1:** Plasma monoamine concentrations and total protein levels in chronic crystal and Captagon users compared with healthy controls.

Parameter	Crystal (*n* = 50)	Captagon (*n* = 50)	Control (*n* = 30)
Dopamine (pmol/mL)	371.76 ± 129.82 (125–590)	345.76 ± 110.48 (154–562)	122.53 ± 55.17 (28–193)
Serotonin (ng/mL)	13.95 ± 1.95 (10.1–19.0)	12.39 ± 0.90 (11.1–15.2)	53.98 ± 2.70 (45.3–59.0)
Norepinephrine (pmol/mL)	186.90 ± 14.18 (129.6–200.5)	189.08 ± 13.10 (129.9–204.0)	118.23 ± 4.08 (112.1–132.1)
Total protein (g/dL)	5.49 ± 0.71 (3.1–7.3)	5.70 ± 0.33 (4.5–6.8)	7.26 ± 0.63 (5.8–8.4)

### 2.3. Gene Expression Analysis (qRT‐PCR)

#### 2.3.1. RNA Extraction and Quality Assessment

The RNeasy Mini Kit (catalog no. 74104, Qiagen, Germany) was used to extract total RNA from PBMCs as per the manufacturer’s protocol. NanoDrop spectrophotometry was used to determine both concentration and purity; acceptable A260/280 ratios were 1.8–2.1 and A260/230 values were 2.0–2.2. Quality control of RNA was performed using an Agilent Bioanalyzer, and only samples with a RIN ≥ 7.0 were used in further analysis.

#### 2.3.2. Complementary DNA (cDNA) Synthesis

cDNA was synthesized from 1 μg of total RNA using the High‐Capacity cDNA Reverse Transcription Kit (catalog no. 4368814, Applied Biosystems, USA) following the manufacturer’s protocol. All reactions were performed in duplicate to ensure reproducibility.

#### 2.3.3. qRT‐PCR Amplification

Quantitative real‐time PCR was performed on an Applied Biosystems 7500 Real‐Time PCR System using PowerUp SYBR Green Master Mix (catalog no. A25742, Applied Biosystems, USA). Primer sequences for SLC6A3, CARTPT, and GAPDH (housekeeping gene) are provided in Supplementary Table S1. Each reaction was performed in triplicate.

Thermal cycling conditions consisted of an initial denaturation at 95°C for 10 min, followed by 40 cycles of denaturation at 95°C for 15 s and annealing/extension at 60°C for 1 min. Melt‐curve analysis was performed to confirm amplification specificity.

### 2.4. Data Analysis and Quality Control

Relative gene expression levels were calculated using the 2^−ΔΔCt^ method (Livak & Schmittgen, 2001). ΔCt values were obtained by normalizing target gene Ct values to GAPDH, and ΔΔCt values were calculated using the control group as the calibrator. Results are presented as mean ± SD. Inter‐ and intra‐assay variability were maintained below 10%.

Supplementary Materials provide all data for the raw Ct and ΔCt of each sample to support transparency and independent verification. Gene expression summary results are provided in detail in Table 2.

**TABLE 2:** Gene expression summary.

Gene	Control ΔCt (mean ± SD)	Stimulant users ΔCt (mean ± SD)	ΔΔCt (users − control)	Fold change (2^−ΔΔCt^)	*p* value
SLC6A3	8.5 ± 1.2	6.0 ± 1.0	−2.5	5.66	< 0.001
CARTPT	9.2 ± 1.3	6.5 ± 1.2	−2.7	6.50	< 0.001

### 2.5. Statistical Analysis

Statistical analysis was completed using Python. Descriptive statistics and boxplots were generated for all parameters. To compare groups, an independent sample *t*‐test or one‐way ANOVA was performed when appropriate. A *p* value < 0.05 was considered statistically significant [5–7].

## 3. Results

### 3.1. Biochemical Marker Analysis

Researchers analyzed the plasma levels of several biochemicals in individuals diagnosed with chronic SUD and found significantly different (abnormal) levels than healthy individuals. Below summarizes the differences found in each biochemical.

**FIGURE 1:**
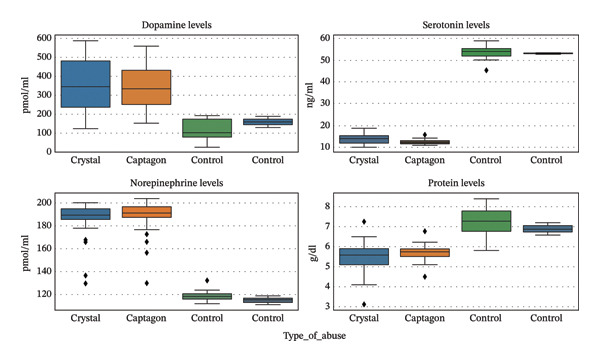
Plasma dopamine, serotonin, norepinephrine, and total protein levels were evaluated in a comparative study of 50 crystal users, 50 Captagon users, and 30 healthy control subjects. The statistical nature of boxplots helps illuminate data; boxes represent the interquartile range, center lines represent the median, and whiskers represent minimum and maximum values. All data were derived from an individual user participant included in the study.

**FIGURE 2:**
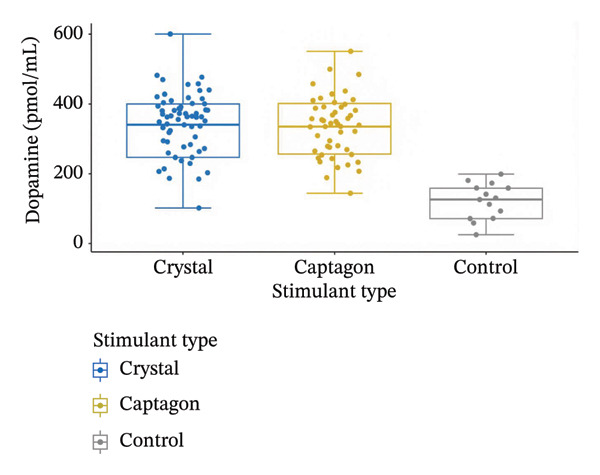
Comparison of plasma dopamine concentrations among crystal users (*n* = 50), Captagon users (*n* = 50), and healthy controls (*n* = 30).

Dopamine (pmol/mL): There were significantly higher dopamine levels in both the meth and Captagon groups than in the control group (Figure 1; Figure 2; Table 1). The mean dopamine levels in the two groups were 371.76 ± 129.82 pmol/mL for meth users and 345.76 ± 110.48 pmol/mL for Captagon users versus 122.53 ± 55.17 pmol/mL for healthy controls. Thus, with more than a three‐fold elevation in dopaminergic activity, chronic stimulant use generates a state of pronounced dysregulation of dopaminergic function. Serotonin (ng/mL): The total amount of serotonin was significantly lower in both the meth users and Captagon users than in the control group (Figure 1; Table 1). The mean levels were 13.95 ± 1.95 ng/mL in the meth group and 12.39 ± 0.90 ng/mL in the Captagon users versus 53.98 ± 2.70 ng/mL in the control group. This substantial depletion of serotonin likely contributes to the affective instability and impulsivity seen in individuals diagnosed with SUD.

Norepinephrine (pmol/mL): The levels of norepinephrine were significantly elevated in both the meth and Captagon groups compared to controls (Figure 1; Table 1). The mean levels of norepinephrine in both groups were approximately 1.585 times higher than the control group (186.90 ± 14.18 pmol/mL and 189.08 ± 13.10 pmol/mL for the meth and Captagon groups, respectively, vs. 118.23 ± 4.08 pmol/mL for healthy controls). Elevated norepinephrine levels indicate increased sympathetic and stress‐related activity. In terms of total protein, there was a reduction in the levels of plasma protein in users of stimulants. The average concentration of protein for users was 5.49 ± 0.71 g/dL for crystal meth and 5.70 ± 0.33 g/dL for Captagon, compared to 7.26 ± 0.63 g/dL for controls (see Figure 1 and Table 1). Lower concentrations of protein may symbolize dietary deficiencies and/or the systemic metabolic consequences of prolonged stimulant use. All biochemical values were significantly different between stimulant users and the control group (all *p* < 0.001), and these shares did remain statistically significant after adjusting for multiple comparisons. Additionally, very large effect sizes were observed with dopamine and serotonin levels of significantly lower than 2.5 for the stimulant users versus those in the control group.

### 3.2. Analysis of Biochemical and Gene Expression Data

The normality of the data was assessed with the Shapiro–Wilk test, and the homogeneity of the variances was tested using Levene’s test and all biochemical variables satisfied the assumptions of parametric testing. The analyses of multiple comparisons through four biochemical parameters and two gene expression studies utilized a Bonferroni‐adjusted alpha level (*α* = 0.0083). The degree of the effect size between stimulant users and controls was large for the difference in the dopamine and serotonin levels as estimated by Cohen’s d.

Reported levels of gene expression due to qRT‐PCR showed a clear biological difference between stimulant users and healthy controls through their comparative analysis. Figure 3 depicts the Log2FC of gene expression levels for 2 genes known to be critical for addiction neurobiology: SLC6A3 (dopamine transporter gene): The Log2FC was +1.98, which would indicate a statistically significant (*p* < 0.05) increase in mRNA levels in stimulant users of ∼5‐fold [6], indicating that there could be a “physiological” increase to the body’s ability to take back dopamine, supporting the development of a tolerance and possibly a diminished response to previously pleasurable stimuli. CARTPT: The Log2FC in CARTPT levels was +2.35, which would suggest that the levels in stimulant users are approximately 7 times higher than healthy controls (Figure 3) [7, 14]; since the CART gene has been associated with stress response and reward, this substantial increase in CARTPT levels may be related to the continued use and/or the compulsive seeking of the abused stimulant. These shifts in transcriptional levels appear to be unique molecular signatures of stimulant exposure and possibly future targets for pharmacogenomic treatment intervention [14–18].

FIGURE 3Changes identified concerning gene expression in user groups based on type of stimulant consumed. (a) Average ± standard deviation of ΔCt value of SLC6A3 in striatum among the following respective user groups: control (i.e., non‐user), alcohol, crystal, Captagon, and polysubstance (mix). (b) Average ± standard deviation of the ΔCt value of CARTPT in the striatum among the same respective user groups. Higher ΔCt = lower gene expression. (c) Comparison of relative gene expression levels as 2^−ΔΔCt^ (fold change) for SLC6A3 and CARTPT of stimulant user groups relative to control. Bars represent average values; statistical significance was determined using a one‐way ANOVA with Bonferroni correction (^∗∗∗^ *p* < 0.001 and ^∗∗∗∗^ *p* < 0.0001).

**Figure:**
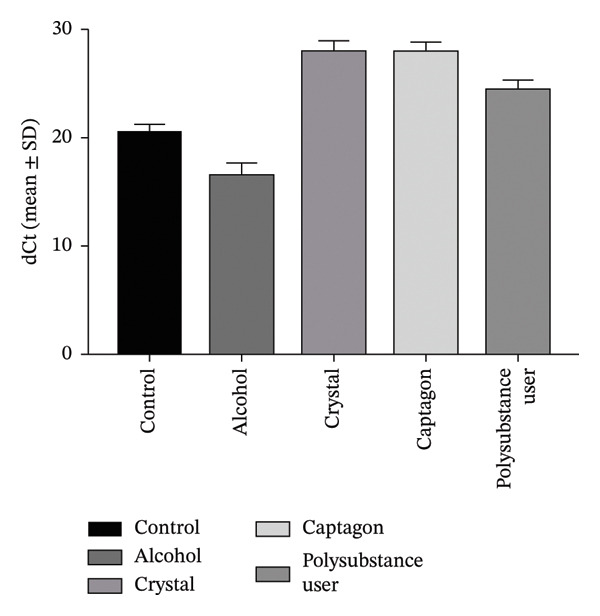
(a)

**Figure:**
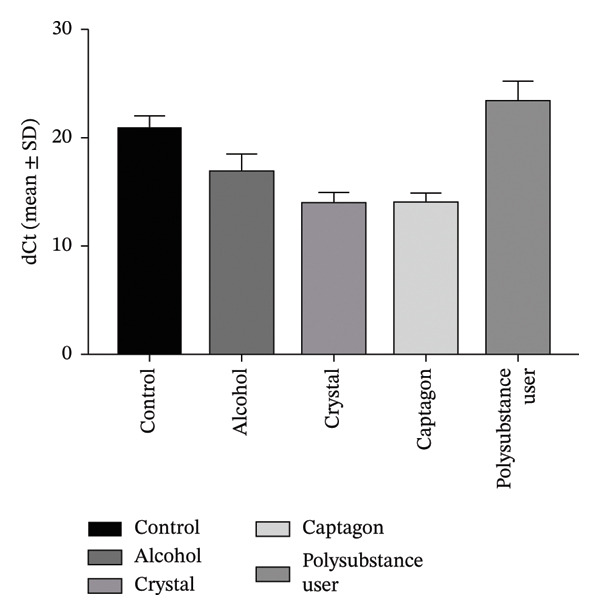
(b)

**Figure:**
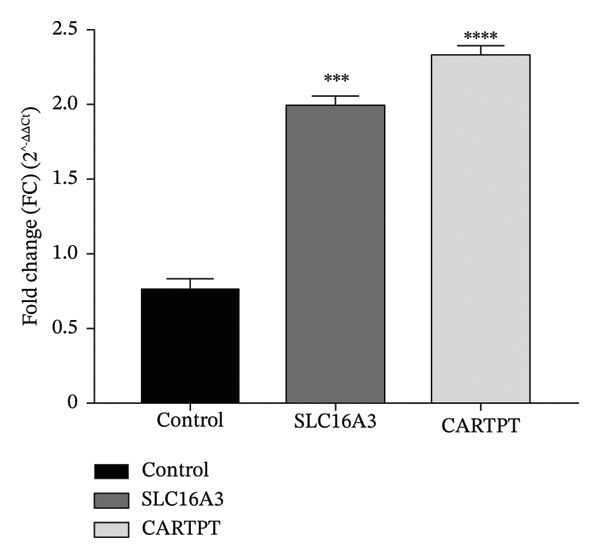
(c)

**FIGURE 4:**
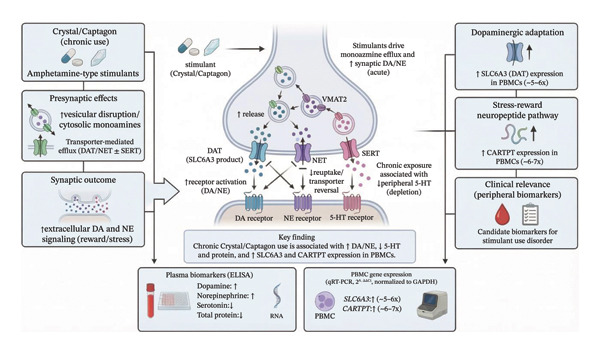
Conceptual model illustrating the neurochemical and peripheral molecular alterations associated with chronic crystal/Captagon use, including monoamine dysregulation and upregulation of SLC6A3 and CARTPT expression in PBMCs.

In order to offer a cohesive interpretation of the biochemical and transcriptional tissue modification seen in users of stimulants, a conceptual model has been devised that illustrates some of the neurochemical and peripheral molecular changes involved (see Figure 4). Repeated exposure to amphetamine‐like stimulants (crystal/Captagon) results in an increase in the extracellular release of monoamines due to transporter‐mediated efflux; therefore, there is an increase in both the concentrations of extracellular dopamine and norepinephrine and a decrease in the availability of serotonin within the brain.

Biochemically, the aforementioned neurochemical changes produce marked increases in the levels of SLC6A3 (dopamine transporter) and CARTPT gene expression in PBMCs, which are consistent with adaptations to the dopaminergic and stress‐reward pathways. Furthermore, because PBMCs also demonstrate decreases in total protein concentrations as well as aberrancies (i.e., alterations to) the profile of monoamines, it can be inferred that there has been a systemic metabolic and neuroendocrine dysregulation due to chronic stimulant use. Overall, these findings suggest that chronic stimulant use will result in coordination of both central and peripheral adaptation and therefore provide evidence for the use of changes in these biochemical and genetic expressions as potential biomarkers for SUD (see Figure 4).

## 4. Discussion

In this study, we show that the use of stimulants has long‐term impacts on brain chemistry and genetics. We are the first to examine monoamines in blood alongside the expression of two key genes (SLC6A3 and CARTPT) in PBMCs amongst users of crystal and Captagon medications. Previous studies have looked at these measures individually or had only examined them in animal models; however, there have been very few studies of the simultaneous or combined effects of these two measures in chronic human stimulant users. Findings from our study indicate that dopamine and norepinephrine levels are dramatically elevated in stimulant users, as demonstrated by biochemical assessments, indicative of increased monoamine activity (the primary way these drugs act) [1]. The results suggest that substances such as crystal and Captagon increase catecholamine availability at the synapse by both enhancing presynaptic release of dopamine and norepinephrine and inhibiting their reuptake via transporter blockade [2]. Notably, the extreme degree of dopaminergic dysregulation identified in our sample, as dopamine levels in both user groups were significantly greater than those within the control group, supports the role of dopaminergic pathways in reward‐related processing and substance‐seeking behavior. This finding is consistent with published literature demonstrating that chronic stimulant use can lead to changes in mesolimbic dopamine neurotransmission, leading to the development of compulsions and tolerance. Users of stimulants have lower levels of serotonin than those who do not use. The serotonin deficit may lead to mood problems, aggressive behavior, and poor impulse control that are impacted by stimulant‐use disorders. The concurrent elevation of norepinephrine evidences hyperadrenergic functioning, which is a contributor to anxiety, hyperarousal, and cardiovascular stress due to chronic use of stimulants. At the genetic level, the differences between stimulant users and non‐users are supported by upregulation of the SLC6A3 gene and CARTPT genes. The dopaminergic gene SLC6A3 was upregulated approximately 5 times in stimulant users, indicating a compensatory mechanism related to dopamine availability when the concentration of dopamine increases in the extracellular space. Increased availability of the DAT may be due to the need to regulate dopaminergic transmission, which results in lower reward sensitivity over time. The CARTPT gene, which encodes the cocaine‐and amphetamine‐regulated transcript peptide, was upregulated approximately 6.5 times in stimulant users versus controls, indicating greater activation of neuropeptide stress‐reward systems. These transcriptional changes likely result from long‐term neuroplasticity associated with chronic stimulant administration. These results suggest that there is a coordinated disturbance in neurotransmitter function (signaling) and gene expression when comparing users and controls. The differences sampled in both the biochemical and molecular analyses are strong indicators of pathology related to stimulant abuse due to the hyperactivity of the dopaminergic system (pressure to continue using) combined with the depressant effects of serotonin (pressure to stop) and the expression of stress‐related peptides. Additionally, SLC6A3 and CARTPT have potential utility as biomarkers and therapeutic targets for treating SUD. It is important to note that the analyses did not account for potentially confounding variables (e.g., smoking status, number of years used and types of substances used, and nutritional status), which could have impacted the biochemical results. The current study’s results should be viewed as generating hypotheses for further study. Although significant changes were observed with respect to peripheral monoamines and gene expression in the PBMCs, these biological measures are an indirect representation by peripheral circulation of CNS neurochemistry. Brain permutations can only be determined by correlating these data to a specific mechanism in the brain; therefore, we cannot make any inferences as to how this may specifically affect CNS neurochemistry or neurotransmission from this data. Future studies should utilize multivariate statistical modeling and longitudinal designs to characterize this and any other associations more accurately.

## 5. Conclusion

The present research illustrates the significant neurochemical/genetic alteration associated with chronic stimulation with drugs like crystal and Captagon. Elevated levels of dopamine and norepinephrine and decreased levels of serotonin and plasma proteins show that chronic use of stimulants leads to severe alterations of both the monoaminergic and metabolic homeostasis among users. The increased expression of both the SLC6A3 and CARTPT genes demonstrates the ongoing reprogramming of the molecular level due to dependence on these stimulants. Furthermore, the alterations in gene expression are consistent with the neurochemical imbalances that have been documented among users and provide insights into the underlying processes involved in addiction‐related behavior. With the integration of biochemical assays and gene expression profiles, this research provides important advancements to the neurobiological substrates of stimulant addiction. By identifying the specific molecular changes that occur with chronic stimulant use, the foundation for future diagnostic and therapeutic approaches has been established and will ultimately lead to the development of personalized intervention strategies aimed at modulating dopaminergic tone and stress‐related pathways. Larger sample sizes, the addition of female participants, and longitudinal follow‐up in future research can provide additional validation of the identified biomarkers and clarification of the recovery process following abstinence. Nevertheless, the current results provide reasonable advancements in addiction neuroscience and underscore important molecular markers associated with SUD. Only male participants were included; no evaluation of sex differences was conducted. No measurements of nutrition or liver function were obtained, which could impact total protein levels. The cross‐sectional design limits the ability to make causal inferences.

## Author Contributions

Yasameen Hameed Jasim conceptualized the study, designed the research framework, and supervised the project. Mustafa Abd‐Almajeed Abd‐Alkareem and Basma A. Al‐Mashhdani conducted the laboratory work and experiments. Rasha Hameed Jasim performed data analysis and statistical evaluation. Sonia Tabasum conducted the literature review and drafted the manuscript. Ashfaq Ahmad Shah Bukhari, Lala Hasanli, and Rajwali Khan contributed to manuscript revision, responses to reviewers, and preparation of the mechanistic illustration.

## Funding

No funding was received for this manuscript.

## Disclosure

All authors have read and approved the final manuscript.

## Ethics Statement

The manuscript does not include animal experiments or human studies; only in vitro studies were used.

## Conflicts of Interest

The authors declare no conflicts of interest.

## Data Availability

The data that support the findings of this study are available from the corresponding authors upon reasonable request.
